# Information and Communication Technology Medicine: Integrative Specialty for the Future of Medicine

**DOI:** 10.2196/42831

**Published:** 2023-07-13

**Authors:** Peter Joseph Jongen

**Affiliations:** 1 MS4 Research Institute Nijmegen Netherlands; 2 Department of Community and Occupational Medicine University Medical Center Groningen University of Groningen Groningen Netherlands

**Keywords:** information and communication technology, ICT, integrative, transdisciplinary, eHealth, internet, medical informatics, application, artificial intelligence, digital medicine, technologies

## Abstract

The impact of information and communication technology (ICT) on medicine is unprecedented and ever-increasing. This has made it more and more difficult for doctors to keep pace with ICT developments and to adequately match the input of ICT experts. As a result, medical disciplines may not be able to take full advantage of growing possibilities. In this personal viewpoint paper, I argue for the establishment of a novel medical specialty, ICT medicine. ICT medicine is needed to optimally face the challenges of ICT-based developments, including artificial intelligence (AI), and to ensure their efficient and beneficial use. ICT medicine is rooted in both medicine and ICT, and in contrast to existing medical specialties it is integrative in nature, as long-standing structural collaborations with ICT and other stakeholders cross the boundaries between disciplines. Thus, new concepts and theories may evolve that are better suited to addressing ICT-related issues in medicine. ICT doctors will be instrumental in the conception, development, implementation, and evaluation of digital tools, systems, and services. They provide a bridge between ICT professionals and clinical users and educate doctors in digital applications and services. Notably, ICT doctors may have a pivotal role in the validation, verification, and evaluation of AI models. ICT medicine institutes offer a home to these new professionals, enhancing their independence within health care organizations and in relation to ICT companies. Importantly, in an era of growing technicalization and use of AI algorithms, ICT doctors may safeguard the human factor in medicine. And, from a societal perspective, they may promote digital inclusion and the continuing high quality of digital services and provide leadership in the future digitalization of medicine.

## Introduction

Information is crucial to the success of medicine. In recent decades, the transmission and exchange of information have been revolutionized by the advent of the internet and ongoing developments in information and communication technology (ICT). At this point, the ICT sector provides a broad range of applications and services for medical use [1]. ICT-based fields, including medical informatics, have emerged in the slipstream of this evolution. Increasingly, collaborations between medicine and the ICT specialties are leading to new diagnostic, prognostic, and therapeutic tools and procedures. Among other consequences, these collaborations should ensure that ICT solutions are designed with all current empirical evidence in mind [2].

However, physicians’ opportunities and capabilities for interacting optimally with ICT specialists tend to lag behind technical developments. For example, a recent review of smart home technologies for health care identified a lack of collaboration across disciplines and noted that technological developments dominate over the human-centric part of the equation [3]. And the implementation of continuous connected care augmented by remote monitoring, which is technically feasible, was found not to be matched by a corresponding shift in clinicians’ approach to the delivery of care [4]. In the Netherlands, general practitioners were early adopters of electronic medical records (EMRs), and they saw the EMR as an instrument for providing evidence-based primary care [5]. However, in terms of ICT-medicine cooperation, the EMR is a rather straightforward use of ICT. At this point, more complex issues are at hand, such as the development and validation of digital biomarkers, the interpretation of automated measurement outcomes and their integrated use in clinical practice, the advancement of connected care models, and the development and evaluation of artificial intelligence (AI)–based algorithms. These current trends place substantially higher demands on the collaboration between ICT and medical experts; therefore, initiatives have a higher risk of not succeeding. Thus, the effectiveness and efficiency of ICT-based solutions may be less than optimal, potentially leading to unexpected challenges and extra expenditures.

Moreover, actual collaborations between medical and ICT professionals are often project-based and ad hoc, whereas implicit differences in perspectives may lead to miscommunication, disagreements, or even the failure of projects. Notably, ICT input is frequently provided by commercial companies whose motives and expectations are potentially at odds with those of medical professionals.

Against the background of both the high potential and the possible pitfalls of medicine-ICT collaborations, appropriate structures are needed to foster optimum cooperation between medical and ICT specialists. As the extensive and radical impact that ICT is having on medical practice and research is unprecedented in history, existing structures cannot be expected to optimally turn possibilities into reality.

In this personal viewpoint paper, I argue for the need for a novel field of medicine: ICT medicine. ICT medicine is needed to optimally face the challenges of new ICT developments, including AI, and to ensure their efficient and beneficial use. It does so by realizing long-standing collaborations between different stakeholders. I argue that ICT medicine—in contrast to existing disciplines—should be integrative in its nature, and I outline the broad range of its activities and the advantages of an institutional organization. Also, in an era of increasing technicalization, ICT doctors may safeguard the human factor in medicine. And, from a societal perspective, they may contribute to digital inclusion and the continuing high quality of digital services and provide leadership in the future digitalization of medicine as a whole.

## Integrative Approach

In recent years, the field of digital medicine has emerged. Digital medicine is concerned with the use of high-quality hardware and software technologies as evidence-based tools for measurement and intervention in the service of human health [6]; given the wide range of stakeholders, its activities are dispersed over a great many disciplines [6,7]. Digital medicine is practiced by the same clinicians and health professionals who practice traditional medicine [7], and, in fact, digital medicine is on its way to becoming just plain medicine [7,8].

Importantly, digital medicine represents multidisciplinary or interdisciplinary collaborations [9]. In the multidisciplinary approach, perspectives, notions, and methods are used that prevail in the respective disciplines, and the end result is essentially a combination of the outcomes of the contributing disciplines. In interdisciplinary cooperation, experts from various fields are enabled to exchange ideas and insights, as a result of which the initial research question may be reframed and new questions may emerge. Yet, as in multidisciplinary research, the questions are formulated in discipline-specific wordings and the problems underlying the questions are perceived within conventional conceptual frameworks.

In contrast to multidisciplinary and interdisciplinary approaches, integrative or transdisciplinary collaborations aim toward insights that emerge by crossing the boundaries between disciplines; eventually these collaborations lead to concepts that may be better suited to addressing the problems at hand [10-14]. Integrative approaches transcend traditional boundaries to integrate various sciences [13-15]. Typically, new hypotheses and theories thrive in the context of integrative collaborations. Notably, “integrationality” may be seen as a mental and intellectual disposition, a habit of mind and behavior toward intentional connection seeking and connection making [13]. Thus, the integrative approach is preferably practiced in a framework of longstanding and structural collaborations with frequent and intensive interactions between researchers from various disciplines. Structural collaborations occur in a coherent organization, such as an institute, where the parts are dominated by the integrative character of the whole [16].

## ICT Medicine

### New Specialty

It has been acknowledged that success in digital medicine requires a fully integrated approach [7]. The existing cooperation between medical and ICT specialists is multidisciplinary and interdisciplinary, often temporary, and focused on selected topics. In order to effectively advance the development and implementation of digital tools and processes it is desirable to facilitate integrative interactions that are enduring and cover all conceivable aspects of ICT. This is best achieved through explicitly and formally integrating the various stakeholders’ input and performance in a new organizational structure. Conceivably, integrative collaborations between medical professionals and ICT experts may materialize as a new specialty, ICT medicine. ICT medicine is both overarching and an integral part of the various existing disciplines. It is rooted in the science and practices of medicine and ICT but goes beyond these fields as its activities surpass barriers between disciplines.

### Activities

ICT doctors cooperate with a wide range of stakeholders, such as physicians, scientists, medical informaticians, medical engineers, data scientists, cyber security experts, ethicists, sociologists, and legal experts, as well as patients and caregivers. ICT doctors initiate, promote, and integrate collaborations in practice and research and are trained to facilitate the development of ICT-based diagnostic, prognostic, therapeutic, and monitoring tools and processes and to identify problems in real-life situations that might benefit from novel ICT-based solutions. As to the latter, it appears that users find it difficult to conceive of or suggest new e-health services that might be useful to them in terms of demand for new services that do not currently exist [17]. ICT physicians will play a key role in the entire trajectory, from idea to innovation. During the design and development phases, they interact with designers, scientists, and practitioners of various medical disciplines, as well as with eventual commercial partners. They will guide scientific assessments and the evaluation of the evidence regarding the intended and unintended effects of ICT-based solutions. In the implementation phase, they will contribute to the education and coaching of the end users and to embedding the tools and services in daily practice. On a continuous basis, they will scientifically evaluate in real-life settings the acceptance, use, effectiveness, and cost-effectiveness of ICT-based changes. And they will educate doctors in digital technology and connectivity. In this way, ICT medicine substantially increases the likelihood that original ideas will transform into widely used cost-effective improvements or, if indicated by the evidence, see to it that provisory “innovations” are altered or discarded.

The integrative approach of ICT doctors may be particularly important in view of the tremendously rapid developments in the field of AI, such as highly flexible, reusable models (foundation models) [18]. The recently proposed generalist medical AI (GMAI) models are expected to be widely applied across medical applications for, among other uses, bedside decision support, augmented procedures, and chatbots for patients [18]. However, like other AI models, GMAI faces critical challenges regarding validation, verification, social bias, and scale [18]. The structural input of ICT doctors on a continuous basis may be invaluable in addressing these issues in terms of supervising the collection and sharing of the vast amounts of medical data that are required, guiding multidisciplinary verification of the input and output, and auditing for inaccuracies, misstatements, and social biases [18].

As adaptive AI algorithms change continuously in response to various types of use [19], it may be necessary with respect to medical AI applications to continuously study the patterns of interaction of doctors and patients with algorithms [19]. ICT doctors should be capable of cocreating and developing, in close collaboration with technologists and other stakeholders, the appropriate languages and methods to evaluate these interactions [19].

And, just as importantly, ICT physicians could be instrumental in facilitating the use of truly open and transparent AI systems in health care, as well as in researching the reproducibility of clinical AI tools, namely those for diagnostic and prognostic purposes.

### Institute

Whereas in multidisciplinary or interdisciplinary collaborations, the various researchers remain employed at their respective organizations and located in their departments, integrative research should be carried out in an institute (an ICT medicine institute) that accommodates all researchers involved and provides them a tenure that complements their activities in other departments. The establishment of ICT medicine institutes also helps to secure the autonomy of ICT physicians, which may be particularly important as ICT-induced changes may at times be revolutionary or disruptive. As an institution, ICT medicine can provide a safe environment for researchers, given that conflicts of interest may arise between the results of an integrative approach and the interests of individual disciplines or commercial partners [20]. In fact, given the unforeseeable consequences of promising technologies like AI and quantum computing, the impact of the activities of ICT medicine is highly unpredictable.

In addition to enabling integrative collaborations with ICT and other experts, ICT medicine institutes may have a coordinating role regarding the often dispersed digital activities in the various departments of hospitals and health care organizations. By so doing they could help not only to prevent an uncontrolled growth of e-health projects and unnecessary duplication, but also promote standardization of and alignment between ICT-based practices inside and outside hospitals. The integration of digital activities between departments would significantly increase their effectiveness and efficiency.

With respect to large-scale ICT systems for public services, including health care, it may be very risky to completely rely on ICT companies. As these companies are shareholder value–maximizing firms, it is highly unlikely that they are objective sources of expertise and competence [21]. Conceivably, given the unique power of big ICT companies through contracts as advisors and vendors, they might ultimately even complicate or hinder ICT-dependent medical innovations [21]. And the updating of systems might be endangered when a company changes policies or ceases to exist. In the end, a dependency on ICT companies may weaken the health care system, as it induces a lack of in-house expertise that is needed for the quality and continuity of core activities [21]. For this reason alone, we should invest in the development of ICT medicine institutes. These institutes can become an independent force that can counterbalance the power of ICT companies. Where appropriate, ICT doctors should be able to cooperate with ICT specialists from public or nonprofit organizations without input from commercial companies, for example, in developing open-source generative AI models [22]. And distinct from commercial companies, ICT medicine institutes may more easily engage medically trained programmers; use programming languages that match the diversity of future users, such as domain-specific languages; and provide user-programmable software [23]. In this way, they may also promote collective digital trust among doctors and patients.

### Human Factor

The impact of ICT on medical research and practice may seriously compromise the human factor in more ways than one. To paraphrase Reiser [24], why seek to inquire into the lives of patients to gain insights into their illness, which not only takes time but is fraught with undependability, if ICT-based techniques and procedures exist that give doctors the ability to identify and quantify clinically relevant signs of disease or changes in these signs by themselves? Thus, the widespread use of ICT, including AI, is set to create a new paradigm of examination and evaluation for the medicine of tomorrow [24,25]. However, technology is not a substitute for engaging with the life of the patient [24,25], and ICT doctors could be the primary defenders of patients’ rights and perspectives.

Given the rapid developments in the field of AI, it is foreseeable that there will be an increasing tendency to eliminate human interference in the design of medical technology and programs and the building of medical and health care systems [26,27]. In the context of the AI-driven technicalization of medicine and health care, ICT doctors could contribute to the protection of the integrity and dignity of the human person and aspects of human values and humaneness [26-28].

Two scenarios are of particular interest in this respect. First, the medicine of the future might be almost completely determined by AI-based automated assessments, diagnostic decisions, and treatment procedures and practiced in AI-designed digitalized health care systems. Second, according to the “One Health” concept, the daily life events of humans are to be comprehensively monitored and analyzed in ICT-integrated environments, such as smart homes, smart cities, and smart hospitals and health care ecosystems [29,30]. In the end, the consequences of these developments could be that doctors and patients find themselves in fully controlled health care systems and that the doctor has transformed from a medical professional into a medical executive and employee.

### Societal Dimension

ICT doctors can also help to ensure that the growing power of ICT-based health care innovations is used appropriately and to facilitate the fair allocation of their benefits [31]. ICT medicine institutes can provide a counterweight to the commercial dimensions of medicine and health care [31]. And in changing political, social, and economic circumstances, ICT physicians may contribute to the quality and continuity of digital medicine and health services [32]. Notably, in the era of ICT, digital inclusion is critical to health care equity; digital inclusion encompasses all activities that ensure that all individuals and communities, including the most disadvantaged, have access to and use of digital services [33]. With respect to medicine and health care, ICT physicians could play a key role in overcoming and preventing structural barriers to digital inclusion relating to age, race, socioeconomic status, language skills, and other factors [34]. They might also see to it that AI-based algorithms are adjusted to local patient populations and health care facilities.

Through vision and by thinking strategically, ICT doctors should be able to lead the way in the field of digital medicine [6,35]. As they have the necessary ICT skills and competence and understand care systems and their complexity, they are very well able to provide leadership in groundbreaking integrative collaborations [36]. Using long-term strategies, ICT doctors may increase awareness among all stakeholders of the potential added value and trust of ICT-based solutions [2]. Importantly, to ensure the implementation and continuity of ICT-based solutions from a financial and administrative perspective, ICT doctors should be able to perform management tasks in health organizations, health insurance companies, and authorities [2].

### Historical Perspective

History shows that technical inventions can lay the foundation for innovations in medicine that have a fundamental and lasting impact on practice routines and patients’ perspectives. Thus, the discovery of a specific type of electromagnetic radiation led to a revolutionary change in diagnostics and the emergence of the discipline of radiology. The increasingly rapid development of ICT-based technologies will be comparable in its dramatic impact. However, the effects of former technologies were limited to certain aspects of medicine, such as diagnosis (radiology) or the treatment of specific patient groups (radiotherapy); basically, the new disciplines complemented the existing ones. In contrast, the current ICT-based revolution pervades *medicine as a whole*, radically changing virtually every aspect of it. These drastic transformations are inadequately structured and formalized in the dispersed activities of digital medicine; they require an overarching specialty that comprehensively and systemically integrates all ICT-based developments and practices throughout all fields of medicine: ICT medicine. With respect to the all-pervasiveness of their impact, ICT developments are comparable to the expansion of microscopic and histological technologies in the 19th century. The latter made cellular pathology the foundation (ie, the infrastructure) of modern medicine, whereas ICT medicine, by operating on a meta level (as a suprastructure) will define the medicine of the 21st century.

## Conclusions

Medicine has entered a period of epochal change. Within a lifetime, age-old practices based on doctors’ individual expertise and collective wisdom are being superseded by knowledge- and evidence-based medicine characterized by, among other factors, automated assessments and AI-driven algorithms. ICT-based tools and processes will be indispensable parts of medical practice, in their role comparable to the preeminent position once held by detailed history taking and physical examination. It can be expected that in the near future, a wide array of ICT-based devices and procedures will be broadly applied by virtually all practitioners in most disciplines. To make this historical transformation a success we must create a new, integrative specialty—ICT medicine.

ICT doctors will not only contribute substantially to the development, implementation, and evaluation of digital tools, systems, and services they will provide a bridge between ICT professionals and clinical users, educate and train doctors in digital medicine, and safeguard the human factor; they may also be leaders in digitized health care organizations [37]. ICT medicine is the specialty that will provide a home for these new professionals [37,38]. Otherwise, we are at risk of having to practice a digital medicine that is both ineffective and costly and therefore poorly accepted by professionals and patients alike.

## Abbreviations

AI: artificial intelligence
EMR: electronic medical record
GMAI: generalist medical artificial intelligence
ICT: information and communication technology
